# Experience of piloting BPaLM/BPaL for DR-TB care at selected sites in Pakistan

**DOI:** 10.5588/ijtldopen.24.0369

**Published:** 2024-11-01

**Authors:** M.A. Khan, A. Ismail, A. Ghafoor, N. Khan, N. Muzaffar, F. Zafar, A. Gupta, S. Foraida, S. Juneja, R. Fatima, A.W. Khan, S. Shahid, M.A. Khan

**Affiliations:** ^1^ Association for Social Development, Islamabad, Pakistan;; ^2^ National TB Control Programme, Islamabad, Pakistan;; ^3^ TB Alliance, New York, USA;; ^4^ Common Management Unit, AIDS, TB, Malaria, Ministry of National Health Services, Regulation and Coordination, Islamabad, Pakistan.

**Keywords:** drug-resistant tuberculosis, integrated care, bedaquiline, pretomanid, linezolid, public hospitals

## Abstract

**BACKGROUND:**

Pakistan ranks fourth globally in terms of high drug-resistant TB (DR-TB) burden, with approximately one-third of cases resistant to fluoroquinolones. Bedaquiline, pretomanid, linezolid and moxifloxacin (BPaLM/BPaL) offers an opportunity for most DR-TB patients to benefit from a shorter, all-oral, well-tolerated and more effective treatment.

**METHODS:**

We conducted a retrospective cohort study to pilot the BPaLM/BPaL regimen at four selected sites in two provinces of Pakistan, i.e. Punjab and Khyber Pakhtunkhwa. Data were extracted and analysed using electronic medical records from the program. Descriptive statistics, survival analysis and binary logistic regression analysis were employed.

**RESULTS:**

A total of 116 patients took treatment between October 2022 and February 2023. The treatment success rate was 96%, with 3% deaths and <1% loss to follow-up. Patients typically completed treatment in 26.2–26.7 weeks for BPaLM and BPaL, respectively. No serious adverse events were observed. The most common side effects included QTcF prolongation (BPaLM: 55%, BPaL: 84%), haematological events (BPaLM: 32%, BPaL: 34%), and gastrointestinal problems (BPaLM: 36%, BPaL: 25%).

**CONCLUSION:**

The BPaLM/BPaL regimens for DR-TB are highly effective with minimal adverse events and feasible to implement in routine program circumstances.

## RÉSUMÉ

**CONTEXTE:**

Le Pakistan occupe la quatrième position mondiale concernant la prévalence de la TB résistante aux médicaments (DR-TB, pour l’anglais « *drug-resistant TB* »), avec près d'un tiers des cas présentant une résistance aux fluoroquinolones. Les traitements comprenant la bédaquiline, le prétomanid, le linézolide et la moxifloxacine (BPaLM/BPaL) permettent à la majorité des patients atteints de DR-TB de bénéficier d'une thérapie plus courte, entièrement orale, bien tolérée et plus efficace.

**MÉTHODES:**

Nous avons réalisé une étude de cohorte rétrospective afin d'évaluer le régime BPaLM/BPaL dans quatre sites choisis au sein de deux provinces du Pakistan, à savoir le Punjab et le Khyber Pakhtunkhwa. Les données ont été collectées et examinées à partir des dossiers médicaux électroniques du programme. Des statistiques descriptives, une analyse de survie ainsi qu'une analyse de régression logistique binaire ont été appliquées.

**RÉSULTATS:**

Un total de 116 patients a reçu un traitement entre octobre 2022 et février 2023. Le taux de succès du traitement s'élevait à 96%, avec 3% de décès et moins de 1% de patients perdus de vue. En moyenne, les patients ont complété le traitement en 26,2 à 26,7 semaines pour les protocoles BPaLM et BPaL, respectivement. Aucun événement indésirable grave n'a été rapporté. Les effets secondaires les plus courants comprenaient l'allongement de l'intervalle QTcF (BPaLM : 55%, BPaL : 84%), des événements hématologiques (BPaLM : 32%, BPaL : 34%) et des troubles gastro-intestinaux (BPaLM : 36%, BPaL : 25%).

**CONCLUSION:**

Les protocoles BPaLM/BPaL pour la DR-TB se révèlent particulièrement efficaces, présentant peu d'effets secondaires, et peuvent être intégrés dans un programme de soins standard.

Multidrug-resistant TB (MDR-TB) poses a significant public health challenge globally, with Pakistan ranking fourth in prevalence worldwide.^1^ Annually, approximately 15,000 new cases of MDR-TB emerge in Pakistan, stressing the urgent need for effective treatment strategies.^2^ Worldwide, efforts have been made to evaluate and implement shorter, more effective treatments that offer improved efficacy and tolerability, especially as conventional options are associated with high rates of adverse events and treatment failure.^3,4^

The emergence of the all-oral 6-month bedaquiline, pretomanid, and linezolid **(**BPaL) regimen, comprising bedaquiline (BDQ), pretomanid (Pa), and linezolid (LZD), has gathered attention as a potential solution to the challenges faced in managing MDR-TB. Initial trials of the BPaL regimen have demonstrated promising efficacy, with a reported treatment success rate of 92% among MDR-TB patients.^5,6^

In Pakistan, where the burden of drug-resistant TB (DR-TB) is substantial, the National TB Control Programme (NTP) has undertaken significant efforts to improve the management of drug-resistant tuberculosis. Programmatic management of drug-resistant TB (PMDT) sites, established under the auspices of the NTP and managed by private partners, serve as essential hubs for the diagnosis and treatment of MDR-TB.^7^ These sites offer free consultation and medication to patients, with more than 3,500 patients with DR-TB treated annually across the country.^8^ Despite these efforts, current treatment regimens for MDR-TB have shown poor treatment outcomes, with reported treatment success rates of 74% and a high incidence of unfavourable treatment outcomes.^9^

Recognising the need for innovative approaches to MDR-TB treatment, the WHO issued a Rapid Communication in May 2022, followed by updated DR-TB treatment guidelines in December 2022, recommending the all-oral BPaL regimen for all eligible DR-TB patients.^10,11^ The rationale behind this initiative lies in the regimen’s demonstrated efficacy, low cost, and potential to improve the quality of life for MDR-TB patients through reduced side effects and shorter treatment duration.^12^

The MDR-TB implementation and care package was adapted and implemented by the NTP and the Association for Social Development (ASD), Islamabad, Pakistan, to include patients on BPaLM and BPaL, based on the WHO operational handbook.^13^ This included a doctor’s desk guide for case management, a training module for physicians and allied staff at PMDT sites, data recording/reporting tools and adverse drug reaction monitoring/reporting guidelines. A 2-day training on updated care protocols and materials was conducted by NTP and ASD for physicians and pharmacists at each PMDT site. This study aims to provide an overview of the implementation outcomes of the BPaLM/BPaL regimen piloted at four PMDT sites in Punjab and Khyber Pakhtunkhwa (KP), Pakistan. Specifically, we present the clinical and programmatic experiences of BPaLM/BPaL implementation, focusing on assessing care outputs, treatment outcomes, feasibility of protocols and guidance for future scale-up efforts.

## METHODS

### Study design

A retrospective cohort study was conducted to evaluate the piloting experience of an all-oral, shorter regimen, i.e., BPaLM (BDQ, Pa, LZD, moxifloxacin) and BPaL at four selected PMDT sites in Punjab and KP. Ethical approval for the study was obtained from the ASD ethical review board (Ref. no. ASD-EAG-23-003).

### Eligibility criteria

The four functioning PMDT sites enabled with Xpert MTB/XDR testing were selected across Punjab and KP provinces. The eligibility criteria for the BPaLM/BPaL regimen included 1) provision of verbal consent, 2) age ≥15 years, and 3) evidence of rifampicin (RIF) resistance without fluoroquinolone (FQ) resistance (for BPaLM) or with FQ resistance (for BPaL). Considering the limited availability of treatment courses, FQ-susceptible patients with extensive lung disease (see definition in Supplementary Data box 2) were prioritised to offer BPaLM.

The exclusion criteria for the BPaLM/BPaL regimen included patients with 1) miliary or TB meningitis, 2) ≥ 1-month exposure or contraindication to any second-line drugs (SLDs) in the regimen, 3) severe baseline comorbid conditions (anaemia: haemoglobin <8 mg/dl, liver condition: lung function test >5 upper limit of normal, heart condition: QTcF>500 ms) and 4) pregnancy.

### BPaLM/BPaL diagnosis, treatment and monitoring

The standard diagnostic and molecular tests to confirm MDR/Pre-XDR-TB, including GeneXpert (Cepheid, Sunnyvale, CA, USA) (RIF resistance), Xpert XDR (Cepheid), phenotypic DST (drug susceptibility testing) and smear/culture tests were conducted for all patients at the time of enrolment. Selected PMDT sites had GeneXpert, Xpert XDR and sputum smear testing facility available on-site, whereas for other sites, samples for sputum culture for TB (BACTEC™ MGIT™ 960; BD, Franklin Lakes, NJ, USA/Lowenstein-Jensen) and phenotypic DST (Becton Dickinson) were transported to national and provincial reference laboratories.^6^ The Xpert^®^ MTB/RIF test (Cepheid) was used to test RIF resistance; Xpert XDR to detect resistance to isoniazid, levofloxacin, moxifloxacin and ethionamide; and phenotypic DST to determine resistance to BDQ, LZD, clofazimine, ethambutol, pyrazinamide, streptomycin, delamanid and pretomanid. The severity of lung disease was determined using chest X-ray, and the findings were recorded as cavitations and the extent of lesions and consolidations.

Patients were assessed for any previous TB history, past exposure to first- or second-line drugs and outcome of previous TB treatment. The following comorbidities were recorded: diabetes, liver/gastrointestinal disease, heart disease, blood disorder, kidney disease, lung disease, immune-compromised conditions, mental health conditions and smoking/alcohol history. Rapid HIV testing, a thorough clinical/ physical examination, and laboratory investigations were done at baseline and presented in Table 1.

**Table 1.** Schedule of examinations during treatment and follow-up phases of the study.

	Baseline assessment and screening	Treatment phase								
Investigation/observation		M1	M2	M3	M4	M5	M6	M7	M8	M9
Demographics, medical history	X									
Informed consent	X									
Clinical examination	X	X	X	X	X	X	X	X	X	X
Treatment adherence		X	X	X	X	X	X	X	X	X
Concomitant treatment	X	(Baseline, then as per comorbidities)								
Adverse events		X	X	X	X	X	X	X	X	X
Sputum smear	X	X	X	X	X	X	X	X	X	X
Sputum culture	X	X	X	X	X	X	X	X	X	X
DST	X									
Haemoglobin/platelets count/white blood count	X	X	X	X	X	X	X	X	X	X
Serum liver enzymes	X	X	X	X	X	X	X	X	X	X
Serum creatinine	X	X	X	X	X	X	X	X	X	X
Serum potassium (at baseline and if clinically indicated or ECG abnormalities)	X									
Pregnancy test (female)	X									
HIV test	X									
ECG	X	X	X	X	X	X	X	X	X	X
Visual acuity and BPNS	X	X	X	X	X	X	X	X	X	X
Chest X-ray	X						X			

M = month; DST = drug susceptibility testing; ECG = electrocardiogram; BPNS = brief peripheral neuropathy screening.

Typically, patients were monitored, and adverse drug reactions, treatment adherence, electrocardiography, treatment response (culture, smear, and clinical indicators) and need-based laboratory investigations during monthly follow-up visits were recorded. Xpert XDR/DST testing was repeated in case of non-response to treatment or temporary treatment interruption (i.e., cumulative interruption between 36 and 59 days or continuous interruption of >35 days) to determine if there is additional acquired resistance.

The standard duration of BPaLM/ BPaL treatment was 6 months, with an extension to 9 months if 1) the sputum culture remained positive after 4 months or 2) after an initial conversion to negative, a single sputum culture reverts to positive. The extension guidelines were according to the National TB Control Programme available at the time of implementation and were updated after this pilot study.

Treatment success was defined as either 1) ‘Cured’, evidence of two consecutive negative sputum cultures taken at least 30 days apart or 2) ‘Complete’, treatment completed for the required duration without evidence of two consecutive negative sputum cultures taken at least 30 days apart but without evidence of treatment failure. Treatment failure of BPaLM/ BPaL was defined as positive sputum culture results of both Weeks 16 and 20 of treatment. Patients were to be re-enrolled on a more extended treatment regimen in case of treatment failure.

### Data collection and analysis

The data of patients recruited from 3 October 2022 until 28 February 2023 on the BPaLM/BPaL regimen were included in this study. Quantitative study indicators were derived from the programme’s Electronic Medical Records (EMR) and patient clinical records on 1 January 2024. Data quality was maintained by verifying it from hard copy data files, ensuring data completeness and correctness and querying any implausible data with the help of MDR program staff.

The analysis was done using SPSS v26 (Statistical Package for Social Sciences; IBM Corp, Armonk, NY, USA). After cleaning, frequencies and percentages were calculated for the categorical variables, while mean and standard deviation were computed for continuous variables. Survival analysis and the Kaplan-Meier method were used to determine the time to conversion of sputum culture. The relationship between multiple baseline covariates and culture conversion at Week 8 was presented as odds ratios.

## RESULTS

A total of 116 patients were enrolled on BPaLM/ BPaL at 4 PMDT sites during the specified period. The baseline characteristics showed that the mean age was 36.9 years, and there were more male patients, i.e., around 63.8%. The average Body Mass Index (BMI) was 18.3 kg/m^2^, 76.5% of the patients were married, and more than half (i.e., 67.3%) of the patients had only studied till primary school. The comorbid conditions at baseline included diabetes (20.7%), anxiety/ depression (15.9%), hepatitis C (0.9%) and epilepsy (0.9%). Other conditions at baseline included smoking (19.8%), anaemia (15.8%) and raised ALT levels (14.3%). None of the patients were found HIV positive at the time of enrolment.

### TB disease characteristics

A total of 118 patients were RIF-resistant on GeneXpert/Xpert XDR; 44 were also found FQ-resistant. Those resistant to RIF only (i.e., 74) were given BPaLM, whereas those with RIF and FQ resistance (i.e., 44) were given BPaL. On phenotypic DST at baseline, two patients (on BPaLM) were FQ-resistant and were shifted to BPaL, and two patients (on BPaL) were found bedaquiline-resistant and were shifted to a longer treatment regimen (LTR).

At baseline, 98.3% of the patients had pulmonary TB, among which 53% had cavitary disease on X-ray chest. 86.9% of patients were acid-fast bacilli (AFB) smear-positive, but culture testing detected *Mycobacterium tuberculosis* (MTB) only in 66.4% of patients. The remaining culture results included 11 (9.5%) culture-negative, 12 (10.3%) contaminated samples, and 16 (13.8%) results not available. As culture results were unavailable at enrollment, the diagnosis was based mainly on rapid molecular GeneXpert test results presented in Table 2.

**Table 2.** Baseline characteristics (*n* = 116).

Characteristics	*n* (%)
Age, years, mean ± SD	36.9 ± 18.1
Age groups, years	
≤25	44 (37.9)
26–44	33 (28.4)
45–64	27 (23.3)
≥65	12 (10.4)
Weight, kg, mean ± SD	50.75 ± 10.6
BMI, kg/m^2^, mean ± SD	18.3 ± 4.7
Sex	
Male	74 (63.8)
Female	42 (36.2)
Marital status (*n* = 98)	
Married	75 (76.5)
Unmarried	23 (23.5)
Education (*n* = 98)	
Years of schooling, mean ± SD	3.8 ± 4.8
Primary (0–5)	66 (67.3)
Secondary (6–10)	23 (23.5)
Intermediate (11–12)	5 (5.1)
Graduate (13–14)	4 (4.1)
Positive HIV status	0
Diabetes mellitus	24 (20.7)
Female	12 (50)
Male	12 (50)
Raised ALT levels (mild) (*n* = 112)	16 (14.3)
Hepatitis C	1 (0.9)
Anaemia (mild and moderate) (*n* = 114)	18 (15.8)
Anxiety/depression (mild and moderate) (*n* = 113)	18 (15.9)
Epilepsy	1 (0.9)
Smoking	23 (19.8)
Drug history	7 (6)
Drug resistance*	
Rifampicin-resistant TB (MDR/RR-TB)	44 (37.9)
Rifampicin- and fluoroquinolone-resistant (pre-XDR-TB)	72 (62.1)
Previously treated with FLDs	90 (77.5)
Outcomes of previous FLD treatment (*n* = 70)	
Failure to previous TB treatment	8 (11.5)
Successful previous TB treatment	7 (10)
Relapse from previous TB treatment	1 (1.4)
Previous TB treatment not evaluated	54 (77.1)
Previous TB treatment with SLDs	0
Site of DR-TB	
Pulmonary only	114 (98.3)
Extrapulmonary only	1 (0.9)
Both pulmonary and extrapulmonary	1 (0.9)
Sub-diagnosis for extrapulmonary	
Skin	1 (50)
Pleural effusion	1 (50)
Total cavitary disease on chest X-ray in pulmonary TB (*n* = 115)	61 (53)
MDR/RR-TB cases	35 (57.3)
Pre-XDR-TB cases	26 (42.6)
Positive sputum AFB smear in pulmonary TB (*n* = 115)	100 (86.9)
Sputum culture	
Positive	77 (66.4)
Contaminated	11 (9.5)
Negative	12 (10.3)
Result not available	16 (13.8)

* Using pre-2021 WHO definitions, MDR-TB was defined as resistance to both isoniazid and rifampicin; pre-XDR-TB was defined as MDR-TB plus resistance to fluoroquinolones.

SD = standard deviation; BMI = body mass index; ALT = alanine transaminase; MDR-TB = multidrug-resistant TB; RR-TB = rifampicin-resistant TB; XDR-TB = extensively drug-resistant TB; FLD = first-line drugs; SLDs = second-line drugs; DR-TB = drug-resistant TB; AFB = acid-fast bacilli.

### Treatment outcomes

The successful treatment rate was 96% for the BPaLM regimen and 95% for the BPaL regimen. Among those successfully treated, the duration of treatment was extended from 26 weeks to 39 weeks for 4 patients (4.3%) on BPaLM and 2 (4.7%) patients on BPaL. The reasons for extension in treatment included 1) lack of culture conversion at week 16 in 3 patients (i.e., 4%) enrolled at BPaLM, and 2) lack of improvement in TB symptoms based on clinical evaluation and X-ray findings at week 26 in 1 (1.3%) patient on BPaLM and in 2 (4.5%) patients on BPaL.

The total number of deaths was 4, of which 2 were being treated on BPaLM and 2 on BPaL. Among those on BPaLM, both patients died within 4 weeks of starting treatment due to extensive lung lesions at baseline leading to respiratory failure. Among those on BPaL, 1 patient died after taking treatment for 10 weeks due to reported elevated α-feto protein levels and hepatic malignancy (i.e., cancer), and 1 patient died due to viral pneumonia leading to respiratory failure after taking 4 weeks of treatment.

Loss to follow-up was minimal, i.e., one patient was on a BPaLM regimen after taking 8 weeks of treatment. However, there was no treatment failure. Overall, the mean time to complete treatment was 26.2 weeks for BPaLM and 26.7 weeks for BPaL regimen, as given in Table 3.

**Table 3.** Treatment outcomes.

Outcomes	BPaLM	BPaL
	(*n* = 72)	(*n* = 44)
	*n* (%)	*n* (%)
Treatment outcomes		
Treatment success	69 (95.8)	42 (95.5)
Loss to follow-up during treatment	1 (1.4)	0
Died during treatment	2 (2.8)	2 (4.5)
Treatment failure	0	0
Treatment success by treatment duration		
Treatment completed within 26 weeks	57 (79.2)	32 (72.7)
Treatment completed in more than 26 weeks	8 (11.1)	8 (18.2)
Successfully completed extended treatment (>26 weeks) based on clinical evaluation and chest X-ray	1 (1.4)	2 (4.5)
Successfully completed extended treatment (>26 weeks) due to non-conversion of culture at Week 16	3 (4.2)	0
Time to complete treatment,* days, mean ± SD	183.6 (9.9)	187.5 (16.2)
BMI at endline, kg/m^2^, mean ± SD	20.1 (5.1)	19.6 (3.5)

* Calculated for patients taken 26 weeks of treatment.

BpaLM = bedaquiline, pretomanid, linezolid and moxifloxacin; BpaL = bedaquiline, pretomanid and linezolid; BMI = body mass index; SD = standard deviation.

The mean time for sputum culture conversion from positive to negative was 6.4 weeks for those on BPaL and 7.6 weeks for those on BPaLM. Among pulmonary TB patients with positive sputum culture at the time of treatment initiation, conversion to negative at Week 4 was achieved in 32 (72.7%) on BPaLM and 21 (63.6%) on BPaL (Figure).

**Figure.**
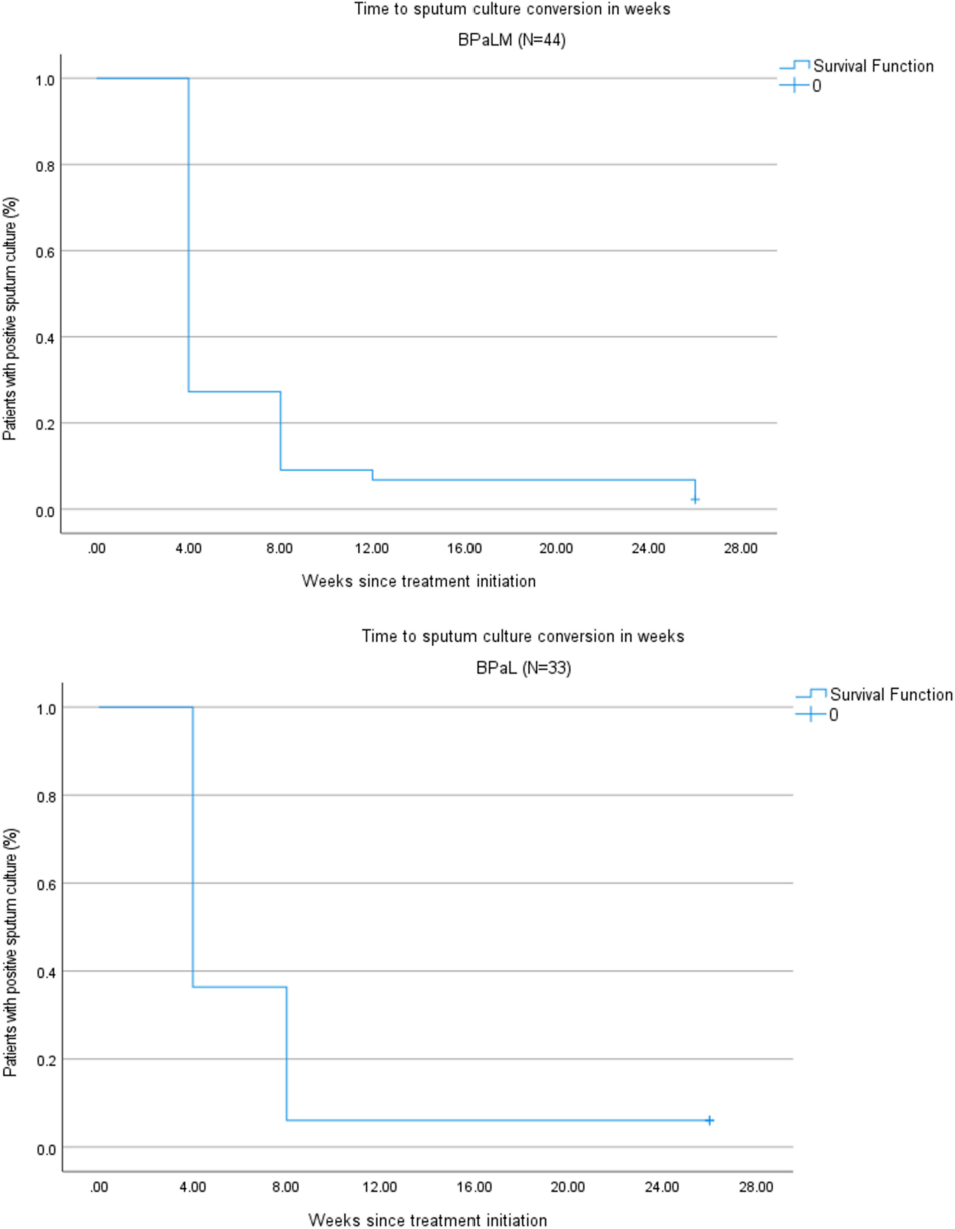
Time to culture conversion in months for BPaLM and BPaL regimens (*n* = 77). BpaLM = bedaquiline, pretomanid, linezolid and moxifloxacin; BpaL = bedaquiline, pretomanid and linezolid.

### Adverse drug events

There were no serious adverse events reported. The most common side effects of both regimens were QTcF prolongation, haematological events, gastrointestinal issues (i.e., nausea, vomiting, diarrhoea, or abdominal discomfort) and neuropathies (i.e. peripheral and optic). Most of the nausea/vomiting was reported during the initial two months, which were treated/ resolved with the addition of anti-gastritis or/and anti-emetics along with anti-TB drugs.

Another common adverse event among patients was elevated liver enzymes. Raised ALT levels were found in 20 (28%) patients taking BPaLM and 10 (23%) patients taking BPaL. However, the condition of these patients improved towards the end of treatment without any change in treatment, as reported in Table 4.

**Table 4.** Adverse drug reactions during treatment with BPaLM/BPaL (*n* = 116).

Events	BPaLM	BPaL
	(*n* = 72)	(*n* = 44)
	*n* (%)	*n* (%)
Haematologic events, i.e., anaemia (Hb < 10.5)	23 (31.9)	15 (34.0)
Severe anaemia (Hb < 8)	0	0
Anaemia requiring LZD dose or frequency adjustment	0	1 (2.3)
Neurologic events	5 (6.9)	4 (9.1)
Peripheral neuropathy^*^	4 (5.5)	3 (6.8)
Optic neuropathy	1 (1.4)	1 (2.3)
Peripheral/optic neuropathy requiring LZD dose or frequency adjustment	1 (1.4)	1 (2.3)
Optic neuropathy requiring LZD discontinuation before treatment completion	1 (2.3)	0
Elevated liver enzymes	20 (27.7)	10 (22.7)
Mild	16 (22.2)	7 (15.9)
Moderate	2 (2.8)	1 (2.3)
Severe	2 (2.8)	2 (4.5)
Drug-induced nephrotoxicity	19 (26.3)	10 (22.7)
Mild	17 (23.6)	9 (20.4)
Moderate	2 (2.8)	1 (2.2)
Prolonged QTcF interval		
Mild	40 (55.6)	37 (84.1)
Moderate	0	2 (4.5)
Other side effects not requiring a change in BPaLM/BPaL regimen		
Gastrointestinal conditions	26 (36.1)	11 (25.0)
Rash or pruritic	1 (3.8)	1 (2.3)
Arthralgia	11 (15.2)	4 (9.1)

* Peripheral neuropathy is measured using a brief peripheral neuropathy screening tool.

BpaLM = bedaquiline, pretomanid, linezolid and moxifloxacin; BpaL = bedaquiline, pretomanid and linezolid; Hb = haemoglobin; LZD = linezolid.

LZD dose adjustment due to adverse events was done in 4 patients (i.e., 2 on BPaLM and 2 on BPaL) out of 116 patients (∼3%). Among the two treated on BPaLM, in one patient with peripheral neuropathy, the LZD dose was reduced to 300 mg daily for 2 weeks (at week 9 of treatment), and in the other patient with significant optic neuropathy, LZD was withdrawn at Week 17. Among the two treated on BPaL, in one patient with anaemia (at Week-9 of treatment), the LZD dose was reduced to 300 mg daily for 2 weeks initially, and due to no improvement in symptoms, the drug was withheld for 4 weeks; and in the other patient with optic neuropathy LZD was withheld for 4 weeks (at Week 9 of treatment), reintroduced at low dose (i.e., 300 mg) for 2 weeks (at Week 13) and finally was put back on 600 mg of LZD daily dose (at Week 15). Although the reintroduction of LZD deviated from the established protocol, it occurred after the reversal of the optic neuropathy, and no symptoms reemerged following the drug's reintroduction.

### Association between comorbid conditions and culture conversion at Week 8

We explored the relationship of multiple baseline variables with the likelihood of culture conversion at Week 8 of both BPaLM and BPaL treatment. The association of culture conversion at Week 8 (in BPaLM and BPaL) with some baseline characteristics showed that extensive lung disease with cavitation (odds ratio [OR] 0.27, *P* = 0.27), being a smoker (OR 0.27, *P* = 0.21), older age (OR 0.98, *P* = 0.51), and people with diabetes (OR 0.27, *P* = 0.21) have lesser odds of culture conversion at Week 8.

## DISCUSSION

This was the first time BPaLM/BPaL had been implemented under routine programme circumstances in Pakistan. Preparations were made, including tailor-made training of selected staff (i.e., doctor and pharmacist) on modified protocols to deliver care and monitor adverse events related to drugs. The training on protocols and quality assurance measures led to better treatment outcomes in people with DR-TB, as similarly experienced in other countries such as Uzbekistan.^14^

Overall, this pilot study demonstrated the protocol feasibility and the potential to achieve higher treatment success among DR-TB patients. The high treatment success rate (i.e., around 95%) of BPaLM/BPaL regimen in both people with MDR and pre-XDR TB is consistent with studies in other parts of the world.^15–17^ The unfavourable treatment outcomes are significantly lower (less than 5%) than the reported outcomes of the longer treatment regimens (i.e., 25% of which, death >16%; loss to follow-up >5%; failure >4%) in Pakistan.

The difference between AFB-positive (86%) and culture-confirmed (66%) results among Xpert-confirmed DR-TB cases needs further attention. Expert consultation identified three plausible explanations for the low culture confirmation *Mycobacterium tuberculosis* (MTB): inadequate sample storage at the treatment facility, poor sample transportation, and inadequate sample handling at the reference laboratory. Similarly, the experience of Tanzania is that ‘no growth in culture’ was mainly due to 1) laboratory technical failures, e.g., improper centrifugation, improper use of reagents for decontamination, and 2) the presence of nonviable bacilli due to inadequate storage and transportation.^18^

In our pilot study, the BPaLM/BPaL regimen achieved ≥60% culture conversion in the first month of treatment. However, in longer treatment regimens, the culture conversion was delayed, i.e., 6% at the end of the first 2 months of treatment and 33% at the end of the first four months of treatment in a study conducted in Karachi.^19^

The reported occurrence of adverse events, through enhanced tailor-made active TB drug-safety monitoring and management (aDSM), may not reflect an absolute measure, as is the case in most programme settings,^17^ but still provides a useful basis for what can be expected when BPaLM/ BPaL gets implemented/scaled under routine programme circumstances. In this pilot study, approximately 34% of patients experienced mild to moderate haematological events, and 9% had neurological symptoms. Experience in Thailand, as reported in a study, was similar: occurrence of haematological events was in 27% and neurological symptoms in 9% of DR-TB patients.^16^

Our piloting study results also found mild–moderate nephrotoxicity in 25% and mild–moderate hepatotoxicity in 27% of DR-TB patients during their treatment span. The occurrence of adverse events highlights the need for regular monthly care monitoring through clinical examination and laboratory investigations.

The strength of this study is that the implementation of BPaLM/BPaL regimens for DR-TB patients was within a routine programme and presents valuable insights into the feasibility and effectiveness of the novel regimen. The utilisation of existing PMDT sites highlights the pragmatic approach taken to pilot these regimens, enhancing the study’s validity and potential for scalability. However, an important consideration missing in this pilot study is evaluating the post-treatment outcomes of patients to understand the sustained effects of the new regimen.

## CONCLUSION

Our findings demonstrate the effectiveness and feasibility of implementing BPaLM/BPaL regimens within routine programme circumstances. This study provides valuable insights into the nationwide scaling of regimens in Pakistan and similar settings in other developing countries.

## Supplementary Material


