# Supplementary material for: Experience of piloting BPaLM/BPaL for DR-TB care at selected sites in Pakistan
Source: IJTLD Open. 2024 Nov 1;1(11):508–15. doi: 10.5588/ijtldopen.24.0369 (PMC11558782; doi:10.5588/ijtldopen.24.0369)
Supplement: Supplementary file 1 [file ijtldopen24-0369_supplementarydata1.docx]

| **Box 1:** Drugs and Daily Dosage   - Linezolid (Lzd): 600mg daily for 26 weeks. If Lzd was to be stopped (due to adverse drug reactions) during the first 9 weeks; then the BPaL/M regimen was discontinued. However, after an initial 9 weeks, the administration of Bdq and Pa continued even when Lzd was discontinued. - Bedaquiline (Bdq): 400mg daily for initial two weeks, then 200mg thrice a week for 24 weeks. If Bdq was to be stopped (due to adverse drug reactions) at any time during the treatment, then the whole regimen was suspended. - Pretomanid (Pa): 200mg daily for 26 weeks. - Moxifloxacin (Mfx): 400mg daily for 26 weeks (if patient found Not resistant to FQ) |
| --- |

# **Experience of piloting BPaLM/BPaL for DR-TB care at selected sites in Pakistan**

| **Box 2**- Extensive Lung Disease Definition:  As per the national guidelines, the extensive lung disease was defined as either:   1. bilateral cavitation or 2. unilateral cavitation >4cm in aggregate or 3. ≥3 lung zones involved either unilateral or bilateral. |
| --- |

| **Box 3**- Cut-offs to determine adverse drug reactions based on laboratory investigations:  **Hypokalemia** (low Serum K):  Mild: 3.4 – 3.0 mmol/L  Moderate: 2.9 – 2.5 mmol/L  Severe: 2.4 – 2.0 mmol/L  Life threatening: <2.0 mmol/L (arrythmia)  **Elevated Liver Enzymes (raised** ALT)  Mild: 1.1 – 3.0 x ULN  Moderate: >3.0 - <5.0 x ULN  Severe: ≥5.0 – 20.0 x ULN  Life threatening: > 20.0 x ULN  **ECG Prolongation** (raised QTcF)  Mild: 450 – 480 ms or up to 30 ms change from baseline  Moderate: 481 – 500 ms or 31 – 59 ms change from baseline  Severe: ≥ 501 ms or ≥60 ms change from baseline without signs/ symptoms of serious arrythmia  Life threatening: ≥ 501 or ≥60 ms change from baseline with signs/ symptoms of serious arrythmia    **Anemia** (low Hemoglobin)  Mild: 10.5 – 9.5 g/dl  Moderate: 9.4 – 8.0 g/dl  Severe: 7.9 – 6.5 g/dl  Life threatening: < 6.5 g/dl  **Nephrotoxicity** (raised Creatinine)  Mild: 1.5 – 2.0 x baseline  Moderate: > 2 – 3 x baseline  Severe: > 3 x baseline  Life threatening: >4.0 mg/dl |
| --- |
